# Sensor system for analysis of biofilm sensitivity to ampicillin

**DOI:** 10.1007/s00253-023-12831-7

**Published:** 2024-01-24

**Authors:** Olga I. Guliy, Stella S. Evstigneeva, Alexander A. Shirokov, Victor D. Bunin

**Affiliations:** 1 Institute of Biochemistry and Physiology of Plants and Microorganisms – Subdivision of the Federal State Budgetary Research Institution Saratov Federal Scientific Centre of the Russian Academy of Sciences (IBPPM RAS), Saratov, 410049 Russia; 2 EloSystems GbR, 13407 Berlin, Germany

**Keywords:** Bacterial biofilms, Antibiotic sensitivity, Ampicillin, Optical system, Electric polarizability, Treatment monitoring

## Abstract

**Abstract:**

The resistance of biofilms to antibiotics is a key factor that makes bacterial infections unsusceptible to antimicrobial therapy. The results of classical tests of cell sensitivity to antibiotics cannot be used to predict therapeutic success in infections associated with biofilm formation. We describe a simple and rapid method for the real-time evaluation of bacterial biofilm sensitivity to antibiotics, with *Pseudomonas putida* and ampicillin as examples. The method uses an electric biosensor to detect the difference between changes in the biofilm electric polarizability, thereby evaluating antibiotic sensitivity. The electric signals showed that *P. putida* biofilms were susceptible to ampicillin and that at high antibiotic concentrations, the biofilms differed markedly in their susceptibility (dose-dependent effect). The sensor also detected differences between biofilms before and after ampicillin treatment. The electric-signal changes enabled us to describe the physical picture of the processes occurring in bacterial biofilms in the presence of ampicillin. The approach used in this study is promising for evaluating the activity of various compounds against biofilms, because it permits a conclusion about the antibiotic sensitivity of biofilm bacteria to be made in real time and in a short period (analysis time, not longer than 20 min). An added strong point is that analysis can be done directly in liquid, without preliminary sample preparation.

**Key points:**

*• Sensor system to analyze biofilm antimicrobial susceptibility is described.*

*• The signal change depended on the ampicillin concentration (dose-dependent effect).*

*• The sensor allows real-time determination of the antibiofilm effect of ampicillin.*

**Supplementary Information:**

The online version contains supplementary material available at 10.1007/s00253-023-12831-7.

## Introduction

To increase the effectiveness of treatment of infectious diseases of various etiologies, one has to ascertain the sensitivity of bacterial biofilms formed in the body to antimicrobial drugs. Currently, the main problem in the treatment of biofilm-associated infections is the lack of an all-purpose method for analyzing biofilm sensitivity to antibiotics and other drugs.

If bacteria are sensitive to a particular antibiotic, it is no guarantee that it will be effective in antibiotic therapy. The insufficient effectiveness of antibiotics is due to the formation of bacterial biofilms, which are extremely resistant to negative factors (Dincer et al. 2020; Romanova and Gintsburg 2011). Up to 80% of human bacterial infections are biofilm associated (Bjarnsholt et al. 2013). Effective therapy of bacterial infections cannot do without the search for new antimicrobials and the detection of their antibiofilm properties in real-time experiments.

Because the existing antibiotics are developed for free-swimming planktonic bacteria, the treatment of biofilm-associated infections does not give satisfactory results. Classical antibiotic susceptibility tests cannot be used to predict therapeutic success in biofilm infections. Because antibiotic sensitivity differs largely between planktonic and biofilm cells, additional variables have been suggested to evaluate antibiotic effectiveness. These include (a) the minimal biofilm inhibitory concentration (MBIC; Thieme et al. 2019), (b) the minimal biofilm eradication concentration (MBEC; Thieme et al. 2019), (c) the biofilm bactericidal concentration (BBC; Macià et al. 2014), and (d) the biofilm prevention concentration (BPC; Macià et al. 2014). Sometimes the MBEC is defined as the lowest concentration of an antimicrobial agent that kills 99.9% of biofilm bacteria, as compared to the control (Dall et al. 2018). Other research groups view the MBEC as being similar to the BBC and relate it to the minimal bactericidal concentration (MBC) of antibiotics for planktonic cultures (Cruz et al. 2018; Macià et al. 2014). The inhibition of biofilm formation by antimicrobials is usually assessed by the MBIC, i.e., the lowest concentration at which there is no time-dependent increase in the average number of viable cells in biofilms. Unlike the MBIC, the BPC is defined as the concentration of an antimicrobial agent at which the initial culture density is decreased so much that biofilms are not formed (Macià et al. 2014).

Currently, many laboratory methods exist to obtain bacterial biofilms and evaluate their sensitivity to antibiotics. In particular, the MBEC and MBIC can be determined by microdilution in 96-well microplates and also in open systems such as biofilm reactors and flow-through cell systems (Macià et al. 2014; Cruz et al. 2018; Thieme et al. 2019; Rafaque et al. 2020). Commercial MBEC testing kits are available, such as the MBEC Assay®, formerly known as the Calgary Biofilm Device (Innovotech, Edmonton, Canada). These, however, have limitations for clinical use, because they do not consider the crossing of the obtained MBEC values with the MBIC ones (Thieme et al. 2019).

Nonetheless, no international standards for the determination of the antibiotic susceptibility of bacterial biofilms have been adopted by official agencies such as the EUCAST or CLSI. Therefore, the development and standardization of new methods for testing the sensitivity of biofilm cultures to antimicrobials is a topical line of research, which in the future will make it possible to ensure effective antibiotic therapy of infections.

Optical and photonic research methods have been repeatedly used to evaluate the effect of antibiotics on planktonic (Blidar et al. 2019; Conteduca et al. 2019; Guliy et al. 2021) and biofilm (Bjarnsholt et al. 2013) bacteria. Most biofilm studies have looked at the mechanisms of biofilm formation and functioning (Yeor-Davidi et al. 2020), the effect of antibiotics and other biocides on biofilm communities (Zhou et al. 1995; Ito et al. 2009), alternative therapies of biofilm infections (Sharma et al. 2019), and methods to control (Estrela and Abraham 2010) and combat biofilm formation (Ben Slama et al. 2013; Mitra 2020). However, no reports have been published on the use of an electric sensor in combination with an optical representation of results to analyze biofilm sensitivity to antibiotics. Table 1 presents comparative data on the main available systems for evaluating biofilm sensitivity to antibiotics. As can be seen, such test systems are few. For example, the method described by Piasecki et al. (2016) evaluates the antibiofilm activity of antimicrobials in real time; yet, it is focused on measuring the viscosity and density of biofilms after antibiotic exposure and accounts for biofilm growth within 4–8 h. Magana et al. (2018) used a commercial MBEC Assay® kit, the Calgary Biofilm Device (Innovotech, Edmonton, Canada), to evaluate the sensitivity of biofilms, and they gave recommendations for monitoring the growth of biofilms and evaluating their viability immediately before antibiotic exposure. In practice, however, this aspect is usually disregarded (Moskowitz et al. 2011; Sandoe et al. 2006; Yau et al. 2015). Further, under actual conditions, it is often difficult to control the formation of biofilms before exposure to an antimicrobial agent, which complicates the interpretation of results. The classical methods for evaluating biofilm sensitivity to antibiotics, although considered the “gold standard,” are laborious and time consuming, taking at least 24 h to complete. Classical antibiotic susceptibility tests cannot be used to predict therapeutic success in biofilm infections. In recent years, laboratory methods have been developed to test biofilm antibiotic sensitivity (Coenye et al. 2018; Hengzhuang et al. 2014; Magana et al. 2018), but most of them are applicable to a small number of clinically important microorganisms. None of them has been accepted as an all-purpose tool to test the sensitivity of biofilms of gram-negative and gram-positive bacteria to antimicrobials of various natures.

**Table 1:** Main available sensor systems for evaluating biofilm sensitivity to antibiotics

No.	Method name	Bacteria	Analysis time	Biofilm control	Reference
1	Diffusion method	all strains	24–48 h	yes	Katongole et al. 2020
2	Microtiter plate method	all strains	24–48 h	yes	Peeters et al. 2008
3	Commercial MBEC testing kits such as the MBEC Assay®			yes	MBEC Assay® 2019
4	Antimicrobial susceptibility testing with the flow cell biofilm model (CLSM). Determination of the MBEC and MBIC by microdilution in 96-well microplates and also in open systems, such as biofilm reactors and flow-through cell systems	*P. aeruginosa*	6 h	yes	Macià et al. 2014; Cruz et al. 2018
5	Microfluidic redox-reactive nanoFET biosensor for extracellular bacterial metabolic analysis	*Bacillus* *subtilis*	600 min	yes	Yeor-Davidi et al. 2020
6	Susceptibility assay on the Calgary biofilm device	*Escherichia coli*, *P. aeruginosa*, *Staphylococcus aureus*	18–20 h for growth and 6 h for analysis	yes	Ceri et al. 1999
7	Piezoelectric quartz tuning fork ring-down system (QTFRS) to measure biofilm viscosity at distinct incubation times *in situ* (Autonomous system for *in situ* assay of antibiotic activity on bacterial biofilms using viscosity and density sensing quartz tuning forks)	*P. aeruginosa*	*in situ* antibiotics are added after 4–8 h of incubation	yes	Piasecki et al. 2016
8	Surface enhanced Raman scattering spectroscopy surfaces with controlled nanometer gap spacings between plasmonic nanospheres for detection of pyocyanin, a secondary metabolite of *P. aeruginosa*, in aqueous media. Nanogaps are incorporated in an in-line microfluidic device, enabling longitudinal monitoring of biofilm formation via pyocyanin detection.	*P. aeruginosa*	3 h after inoculation and quantification in under 9 h	yes	Nguyen et al. 2018
9	Luminescent nanosensors for ratiometric monitoring of three-dimensional oxygen gradients.	*P. aeruginosa*	20–90 min	yes	Jewell et al. 2019
10	Thermal sensor system, thermal waves are generated by a chromium heater, excited with a sinusoidal signal (40 Hz). The resulting temperature changes are detected by an amorphous germanium thermistor. To determine the thermal properties of fluids, the amplitude variation and phase shift of the measured temperature oscillations over time are analyzed. White light interferometry was used for optical control to determine the amount of bacterial adhesion	*E. coli* and *S. aureus*	Real-time 2–24 h	yes	Wieland et al. 2021
11	Novel AlpB/colloidal gold (CG)/nanoporous gold (NPG)/Nafion-reduced graphene oxide (rGO)/glassy carbon electrode (GCE) biosensor on the basis of the heterologous expression of AlpB.	*Helicobacter pylori*	Rapid screening	yes	Xiao et al. 2022
12	Monitoring extracellular electron transfer on a 3-D paper-based cell culture platform	*P. aeruginosa*	Real-time 96 min	yes	Rafiee et al. 2022
13	Optoelectronic device based on a dual array of interdigitated micro- and nanoelectrodes in parallel, aiming at monitoring the bacterial biofilm evolution by using optical and electrical measurements	*E. coli*	Real-time	yes	Brunetti et al. 2021
14	А threshold-activated feedback-based impedance sensor-treatment system for combined real-time detection and treatment of biofilms	*E. coli*	Real-time	yes	Subramanian et al. 2017
15	Flexible platform consisting of gold interdigitated electrodes patterned on a polyimide substrate for *in situ* impedimetric detection and bioelectric effect treatment of biofilms	*E. coli*	Real-time	yes	Huiszoon et al. 2019
16	Electric analytic sensor system based on a hybrid approach that combines the effect of an electric field on the sample under study with an optical result	*P. putida*	Real-time (less than 20 min)	no	Present study

Here we developed a test system for evaluating biofilm sensitivity to antibiotics in the shortest possible time. We used a hybrid approach that combined the effect of an electric field on the test sample with an optical representation of results obtained by recording changes in the electric polarizability of bacteria. Because bacteria react to antibiotic action by changing their morphology, metabolism, and ionic and macromolecular compositions (Mitosch and Bollenbach 2014), these processes, affecting each other, are accompanied by a redistribution of ions and charges. This redistribution can be detected with an electric sensor by measuring changes in electric polarizability.

## Materials and methods

### Strain and culture conditions

*Pseudomonas putida* TSh-18 (IBPPM 358) was from the IBPPM RAS Collection of Rhizosphere Microorganisms (http://collection.ibppm.ru). For biofilm formation at the air–liquid interface, bacteria were cultured in the Luria–Bertani (LB) medium (Bertani 1951) in Erlenmeyer flasks under stationary conditions at 30°C for 4 days.

### Phase-contrast microscopy

Phase-contrast microscopy before and after biofilm treatment with ampicillin and measurements of biofilm thickness were conducted on a Leica LMD 7000 laser microdissection system (Leica Microsystems, Germany) at the Simbioz Center for the Collective Use of Research Equipment in the Field of Physical–Chemical Biology and Nanobiotechnology (IBPPM RAS).

Biofilms were stained with aqueous 1% crystal violet, as described by O’Toole and Kolter (1998). They were separated from the planktonic culture with a coarse-mesh nylon filter, placed on a cover slip, moistened with a 1% crystal violet solution, and incubated at room temperature for 30 min. The dye was then removed, and the samples were carefully washed with a phosphate buffer until the washing solution became completely clear. The stained biofilms were dried at 50°C and subjected to phase-contrast microscopy at 40× magnification.

For biofilm thickness measurements, samples were prepared and analyzed as recommended by Shelud’ko et al. (2015). Before microscopy, cover slips with applied biofilms were turned upside down and placed on concave glass slides. For measurement of biofilm thickness, the microscope was first focused on the “lower surface” of the biofilm and the focal length was recorded in μm (*Z*_1_). Next, the microscope was focused on the “upper surface” of the biofilm and the focal length was recorded in μm (*Z*_2_). The biofilm thickness was calculated from Eq. (1):

1 $$Z=\left({Z}_2{-}{Z}_1\right)\times \left({n}/{n_1}\right),$$

where *n* is the refractive index of glass (1.5) and *n*_1_ is the refractive index of air (1.0).

The determination of biofilm thickness had no fewer than 15 replications.

### Confocal laser scanning microscopy

For confocal microscopy, a TCS SP5 confocal laser microscope (Leica Microsystems, Germany) was used. Signals were recorded by using an argon multilinear laser with an excitation band of 488 nm (power, 20%) and a helium–neon laser of 543 nm (power, 20%). Emission was recorded at 496–506 and 553–580 nm. The magnification of the oil immersion lens was ×100. Scanning was done with resolutions of 1024 × 1024 and 2048 × 2048 and with a digital magnification of ×1000–×3000. All microscopic experiments used equipment provided by the Simbioz Center for the Collective Use of Research Equipment in the Field of Physical–Chemical Biology and Nanobiotechnology (IBPPM RAS).

Images were obtained with Leica LAS X software (Leica, Germany) and were analyzed with Fiji software (open source image processing software) and Vaa3D software (open source visualization and analysis software).

Biofilms were stained with SYTO 9 dye and with propidium iodide (LIVE/DEAD BacLight™ bacterial viability kit; Thermo Fisher Scientific, USA). Live portions fluoresced green, and membrane-compromised portions fluoresced red (Robertson et al. 2019).

### Determination of biofilm biomass accumulation

Before biofilm staining, the planktonic cultures were taken from the flasks, and on addition of aqueous 1% crystal violet, they were incubated at room temperature for 30 min. The dye was then removed and the flasks were washed with a phosphate buffer. For determination of biofilm biomass, the dye was dissolved in ethanol and the absorbance of the solution was measured at *A*_590_ (*l* = 0.5 cm).

### Bacterial viability test

Colonies formed from individual viable cells after exposure to ampicillin (0.5, 1.0, 2.5, 5.0, 10, 25, 50, 100, 250, 500, and 600 μg/mL) were counted by the standard method of plating on solid nutrient media (Methods 1998). The cells grown without ampicillin were used as the control. After the cells had been grown in a thermostat at 30°C for 24 h, the number of colonies was counted.

### Sample preparation and optical analysis of cell suspensions

After the biofilm had been separated from the planktonic culture, the cells were sedimented by centrifugation (13000 × g, 5 min), washed once, and suspended in sterile distilled water. When measurements were made on a Specol photoelectron colorimeter (Carl Zeiss, Germany) at 420 nm, the absorbance of the cell suspensions was adjusted to 1.0.

**Fig. 1:**
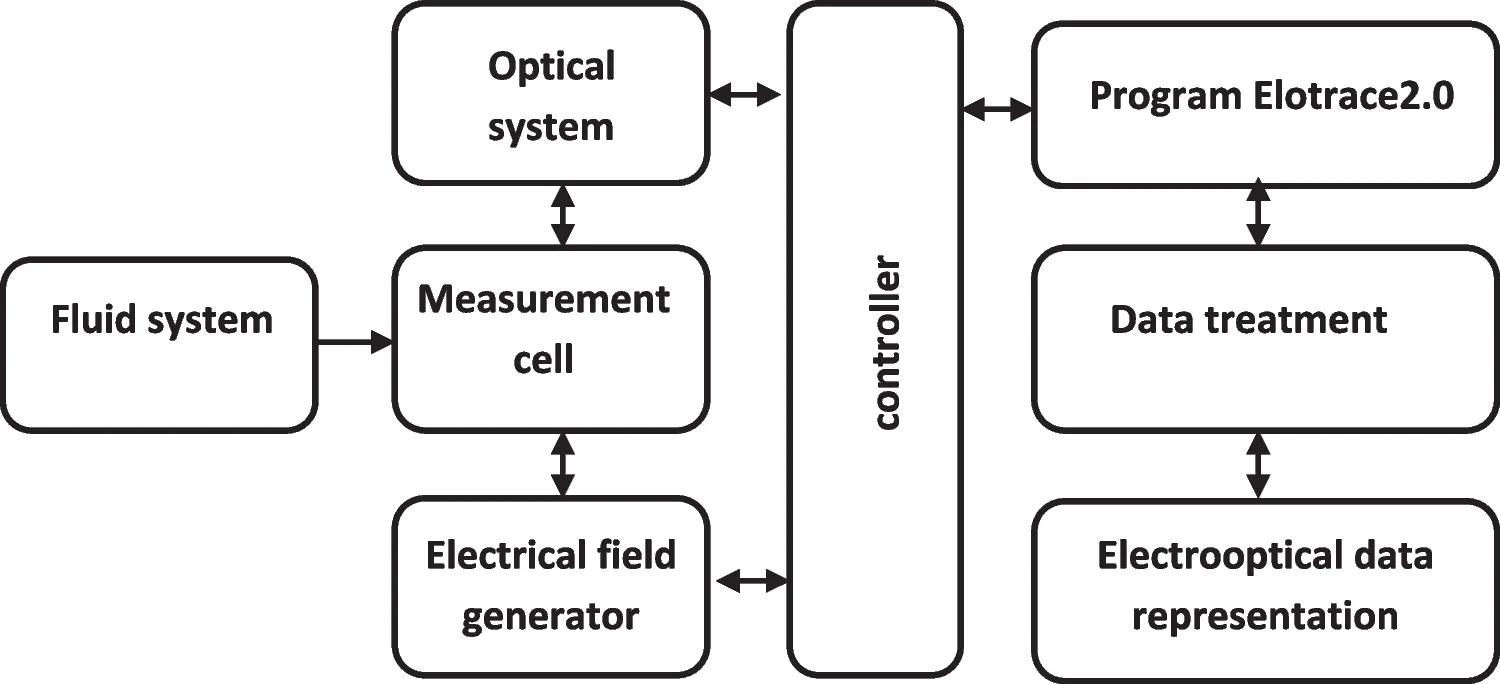
The general scheme of the sensor system

Measurements were made with an Elotrace 2.0 measurement unit (EloSystem GbR, Germany). Figure 1 shows the general scheme of the sensor system. The electric field strength was 90 V/cm, the light wavelength was 670 nm (relative to vacuum), the field application time was 3.0 s. the volume of the measuring cell was 1.0 mL, and the orienting-field frequencies were 700–2800 kHz. Although the Elotrace 2.0 unit enables an automatic algorithm of sample preparation to be implemented, samples were prepared manually.

The Elotrace 2.0 unit ensures the generation in the suspension under study of a harmonic electric field of specified frequency, voltage, and duration of exposure. These variables can be programmed to be changed. The sensitivity of bacteria to various effects manifests itself as changes in the polarization properties and leads to different degrees of cell orientation. The relative measurement error is reduced to 3%.

The polarization and morphometric variables of the cells after ampicillin effect on the sample under study were determined by measuring the optical properties of the suspension. Figure S1 shows the main scheme of the sensor measurement before and after antibiotic treatment.

Each set of optical experiments had no less than five measurements. Data were analyzed with Excel 2010 software and with standard methods of statistical data processing.

### Statistical analysis

Results are presented as mean ± standard error of the mean (SEM). Differences from the initial level in the same group were evaluated by the Wilcoxon test. Intergroup differences were evaluated by the Mann–Whitney test and by ANOVA-2 (post hoc analysis with Duncan’s rank test). For all analyses, the significance levels were set at *p* < 0.05.

## Results

### Theoretical aspects of sensor-based measurement

During biofilm formation, the physiological state of bacteria undergoes changes, and so may their sensitivity to antibiotics (Ito et al. 2009; Otto 2008). A change in the physiological state of biofilm cells leads to a variation in their electrophysical variables, which can be recorded by an electric sensor. The operation principle of the sensor is based on the analysis of the polarization characteristics of biofilms. Biofilm cells are probed with an alternating electric field of intensity *E*, and the electric characteristics of cells associated with a change in their electric polarizability are measured. The polarizability tensor describes the distribution of charges along the cell structure interfaces. The mechanism by which induced charges are formed is rather complicated (Bottcher 1982). For practical purposes, however, it is enough to know that the tensor of axisymmetric particles, to which *P. putida* TSh-18 belongs morphologically, has a longitudinal and a transverse component. Because the value of the complex electric conductivity of the cell structure material includes the electric field frequency, and because the dielectric constant of the cell structure material weakly depends on it, the integral parameter will have frequency dependence (dispersion). As a result, the sum of the induced charges will be determined by the ratio between the properties of two adjacent pairs: cytoplasm–membrane and membrane–external environment.

Determination of the difference Δγ(ω) between the longitudinal γ_а_(ω) and transverse γ_b_(ω) components of polarizability becomes possible because the interaction of the induced charges with the field changes particle orientation and manifests itself as a change in the optical properties of the suspension. The dependence of δ*ОD* on the orienting-field frequency coincides with the frequency dispersion of the anisotropy of the particle polarizability tensor Δγ(ω)=γ_а_(ω)-γ_b_(ω) = K*δ*ОD/OD* (where *OD* is the optical density of the cell suspension) with an accuracy of the constant K.

**Fig. 2:**
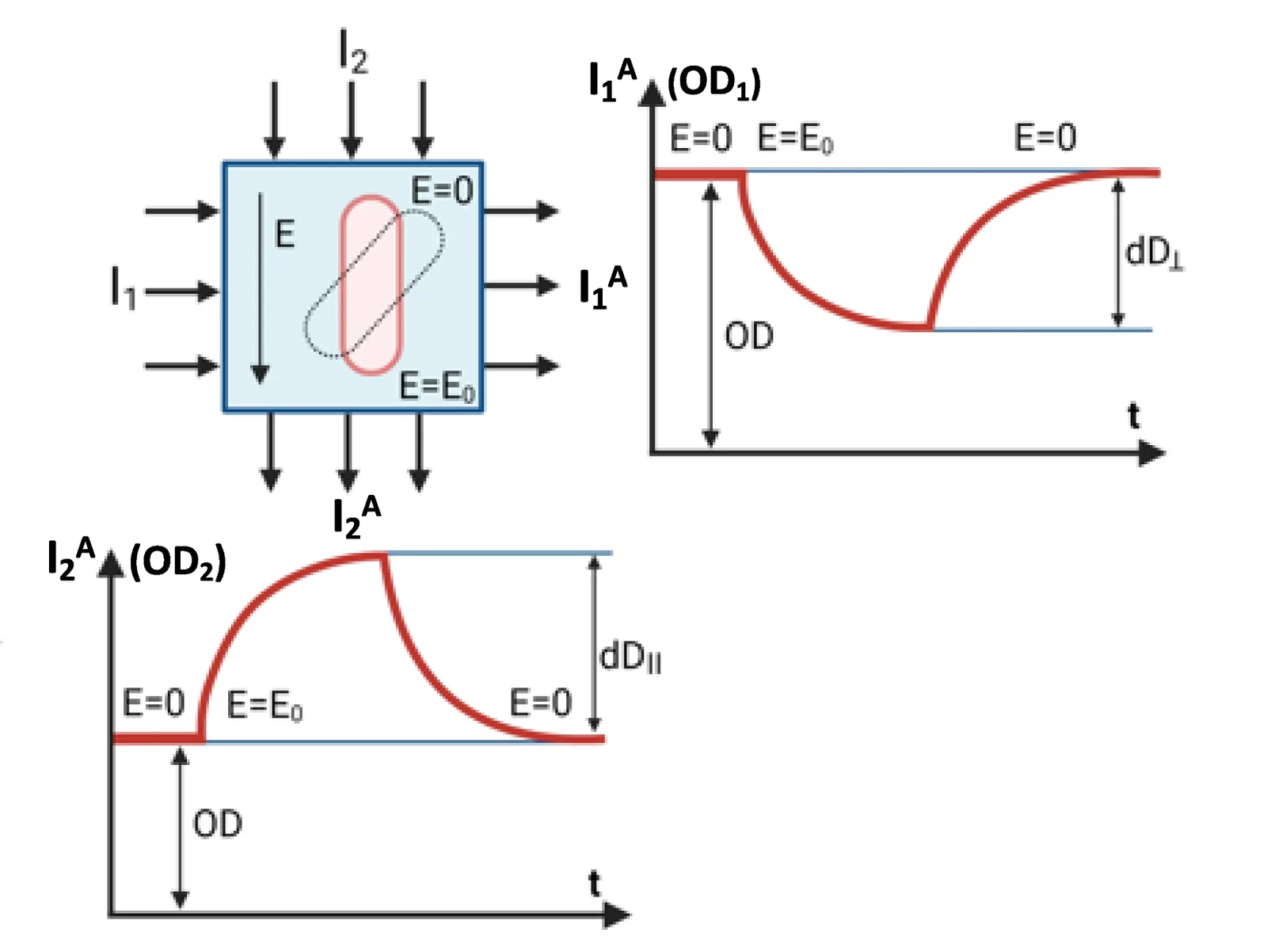
The relationship between action of the electric field and changes in the optical density of the suspension during measurement

The normalization of δ*ОD* to the *OD* value of the suspension at a constant strength of electric harmonic field strength affords an absolute criterion for the change in cell polarizability [Δγ(ω)=K*δ*ОD/OD*]. One can thus comparatively estimate the change in the electric properties relative to the control samples when the cells are exposed to the antibiotic. Figure 2 shows the relationship between action of the electric field and changes in the absorbance of the suspension during measurement.

Analysis of the polarizability anisotropy plot allows one to find the electric properties of individual cellular structures. Measurement of the polarizability anisotropy in the frequency range 600–800 kHz makes it possible to determine the electric conductivity of the cytoplasm. Measurements in the frequency range 1.0–4.0 MHz allow calculation of the transport activity through the cytoplasmic membrane. Measurements in the frequency range 20–200 kHz make it possible to determine changes in the electric properties of the cell surface. In turn, the determination of the specific conductivity of the cytoplasm through the polarizability anisotropy enables one to find the concentration of mobile ions in the cytoplasm. There is a linear relationship between all these variables, with constant coefficients of conversion of one quantity to another.

The second quantity of the electro-optical signal is the normalized area S_2_ under the curve of cell transition from oriented to random state. Figure S2 shows the changes in the electro-optical signal in cell orientation (*S*_*1*_) and disorientation (*S*_*2*_)—the parameters used for calculations. For the subsequent calculations of the morphometric variables, we chose the normalized parameter *S*_*rel*ax_, which is determined by formula (2) and permits calculation of the average cell size:

2 $${\boldsymbol{S}}_{\boldsymbol{relax}}=\left({\boldsymbol{S}}_{\textbf{1}}+{\boldsymbol{S}}_{\textbf{2}}\right)/{\boldsymbol{S}}_{\textbf{2}}$$

Table S1 shows the coefficient values for the polynomial function for calculating the average cell size on the basis of *S*_*relax*_ on the selected time scale *T*.

An electric field in the frequency range 10–500 kHz penetrates the cells and allows indirect determination of the electric conductivity of cellular structures by changes in the optical properties of the suspension (ΔOD). Here we used the connection of the polarization of the boundaries between cell structures exposed to an electric field with the degree of orientation of the cells relative to the field vector direction (see Figure S3). The greater is the difference between the electric conductivities of the neighboring structures, the greater is the effect of these differences on cell orientation and, consequently, on the optical properties of the suspension.

For determination of changes in the state of the cytoplasmic membrane, the results of measurements at 2100 kHz were used. The electric conductivity of the cytoplasm is proportional to the metabolic activity of the cells (Junne et al. 2010; Guliy and Bunin 2020).

To measure the viability of the biofilm cells exposed to ampicillin, we used the linear correlation of this quantity with the optical signal magnitude. For calculations, we used the formula given in Guliy et al. (2021). Pearson’s correlation coefficient was 0.9, which is a strong correlation, because Pearson’s coefficient is ≥ 0.7.

### Detection of biofilm antibiotic susceptibility by sensor system

**Fig. 3:**
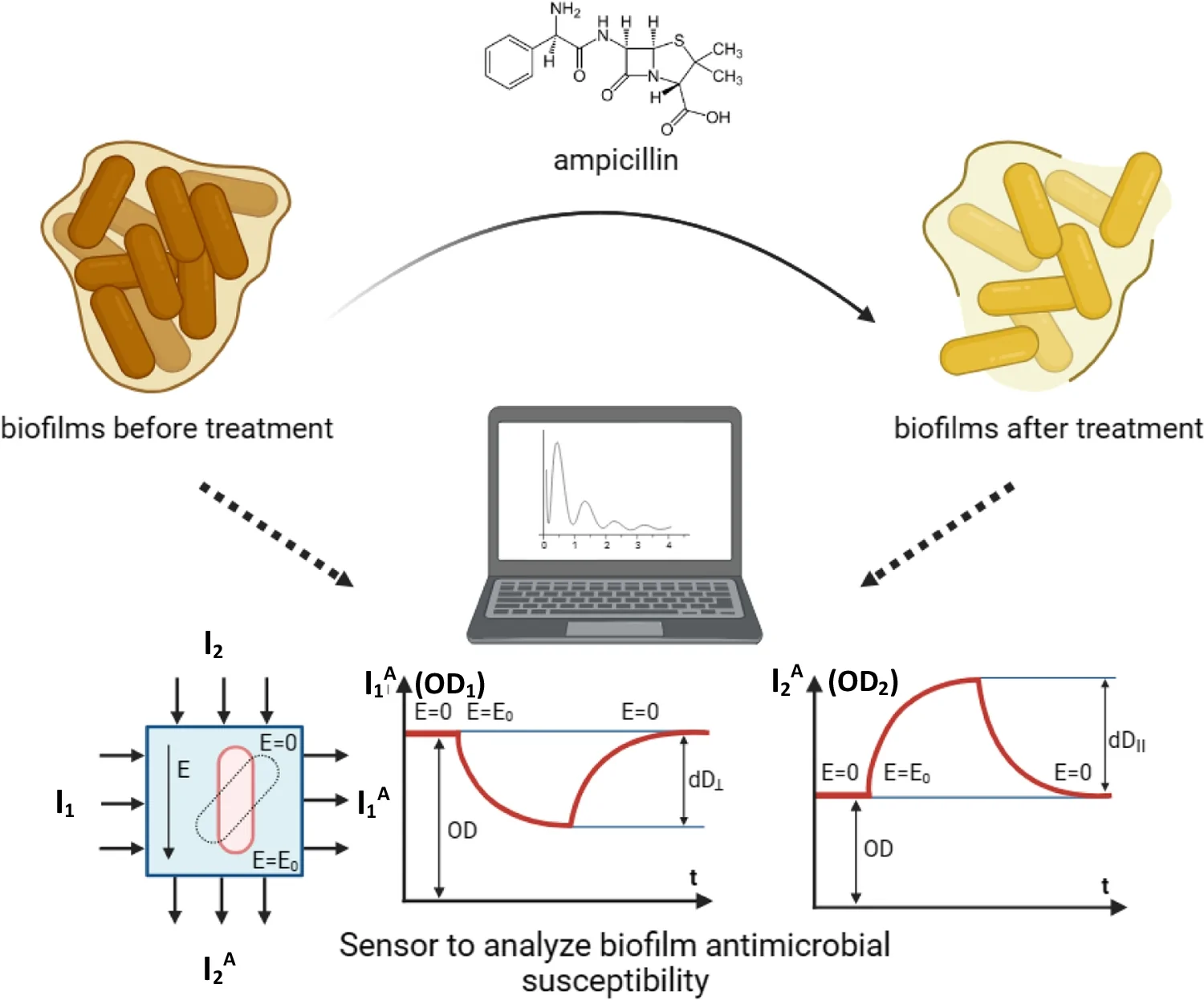
The general experimental scheme (created with BioRender.com)

*Pseudomonas* bacteria are clinically important because they can form biofilms—complex bacterial communities that are highly tolerant of antibiotics (Smirnova et al. 2010). Here we used the gram-negative bacterium *P. putida* TSh-18, which is capable of forming biofilms, and ampicillin, which belongs to β-lactams, one of the most numerous groups of antibiotics (Sales of veterinary antimicrobial agents in 31 European countries in 2018 2020). Figure 3 shows the general experimental scheme.

Several research groups (Brady et al. 2017; Dall et al. 2018) have recommended that the antibiofilm action of ampicillin be evaluated on the basis of data from biofilms grown under standard, no-antibiotic conditions. The structure of biofilms (number of viable cells, total biofilm biomass, biofilm thickness, etc.) is assumed to be stable until the antibiotic directly acts on it. In our studies, therefore, analysis of changes in the signal magnitude, determination of CFU/mL, and phase-contrast microscopy were done with ampicillin-treated and untreated biofilms. This was followed by comparison of the obtained data.

A key point in analyzing antibiotic effects on biofilms is to measure biofilm thickness (Macià et al. 2014). As recommended by the manufacturer of the commercial MBEC Assay® kit, biofilm growth should be monitored before antimicrobials are used (MBEC Assay® 2019).

In most studies, however, the antibiofilm effect of antibiotics has been detected in the absence of uniform standards of biofilm growth (Moskowitz et al. 2004, 2011; Sandoe et al. 2006; Yau et al. 2015). In this study, before the sensitivity of *P. putida* TSh-18 biofilm to ampicillin was tested, the biofilm thickness was measured. On day 4 of growth, the biofilms had the maximal thickness (25.7 ± 1.3 μm on average), which indicated that they were mature. In subsequent work, the effect of ampicillin on biofilms was examined by growing the bacteria under the same conditions until biofilms attained maximal thickness.

We next measured the magnitude of the optical signal from the biofilms of *P. putida* TSh-18 exposed to ampicillin concentrations between 0.5 and 600 μg/mL. The choice of these concentrations was based on the data generated by standard microbiological plating of ampicillin-exposed cells and on the earlier results for planktonic cells of *P. putida* TSh-18 (Guliy et al. 2021). The difference between the values of the optical characteristics before and after exposure to the antibiotic served as an analytic signal.

**Fig. 4:**
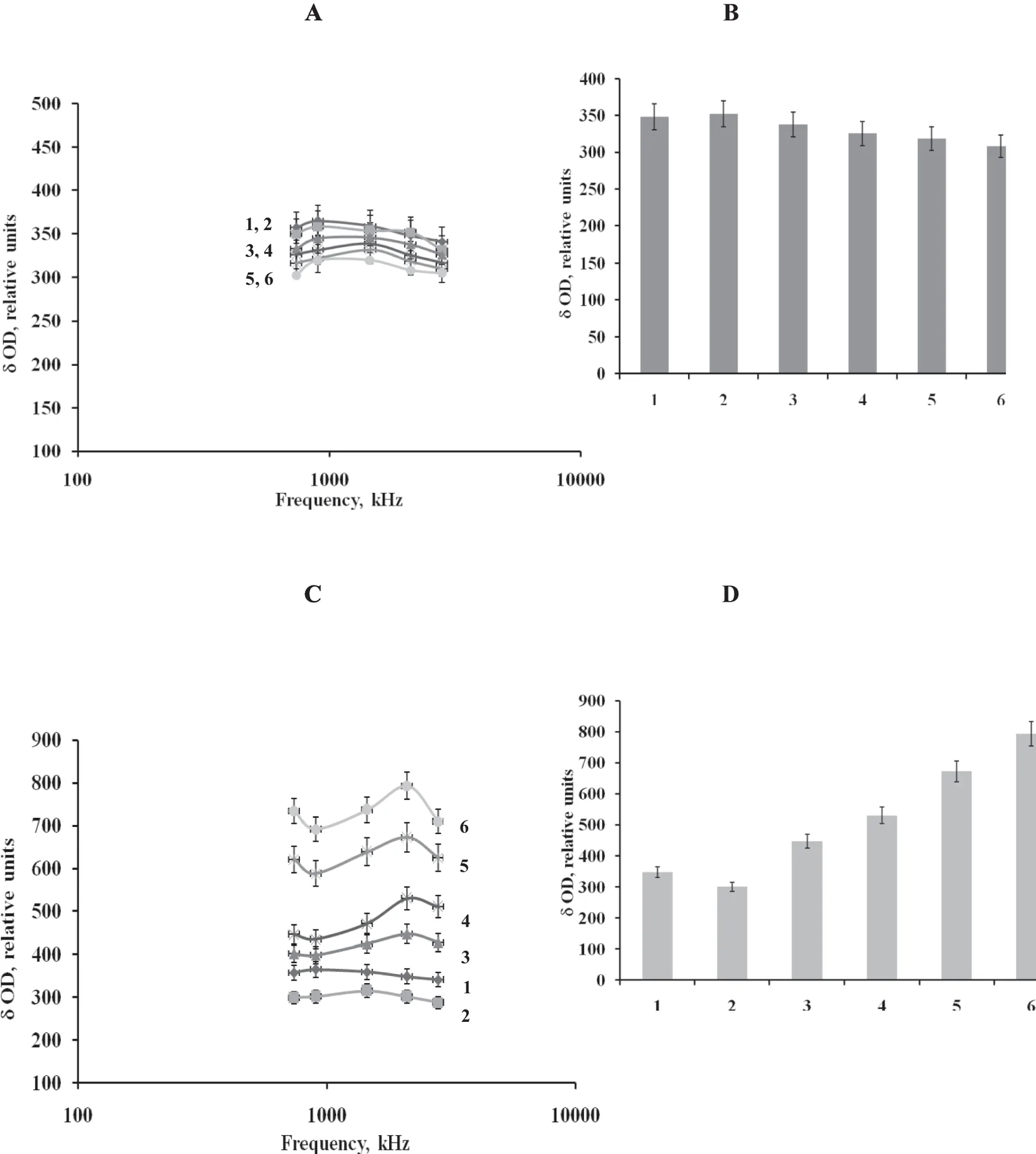
(A) Change in the magnitude of the analytic signal from a biofilm suspension of *P. putida* TSh-18 versus the amount of ampicillin. *1*, control (no antibiotic); *2*, 0.5; *3*, 2.5; *4*, 5; *5*, 10; *6*, 25 μg/mL of ampicillin. (B) Change in the polarizability anisotropy value at 2100 kHz. (С) Change in the magnitude of the analytic signal from a biofilm suspension of *P. putida* TSh-18 versus the amount of ampicillin. *1*, control (no antibiotic); *2*, 50; *3*, 100; *4*, 250; *5*, 500; *6*, 600 μg/mL of ampicillin. (D) Change in the polarizability anisotropy value at 2100 kHz

Figure 4A shows images of biofilm cells before and after exposure to 0.5–25 μg/mL. When 0.5–10 μg/mL of ampicillin were used, changes in the magnitude of the signal from biofilms were no greater than 5–7%. The invariability of the shape of the signal spectra characterizes the absence of morphometric changes in the cells. The linear relationship between the magnitude of the signal and the electric conductivity of the cytoplasm at frequencies between 500 and 900 kHz makes it possible to use the results at any frequency that lies in this range. For convenience of presentation, Fig. 4B shows the data obtained at 2100 kHz (change in the analytic signal value at 2100 kHz). From these data, it can be seen that with 25 μg/mL of ampicillin, the changes in the signal magnitude (control minus experiment) were ~11–12%.

We next measured the optical characteristics of the biofilms at greater ampicillin concentrations (50–600 μg/mL) (Fig. 4C). At 50 μg/mL of ampicillin, the signal magnitude decreased by 17%, as compared to the no-antibiotic control (Fig. 4D). With a further increase in the ampicillin concentration, the optical signal magnitude increased, peaking at 600 μg/mL of ampicillin.

An additional informational parameter in the analysis with biofilm bacteria exposed to an antibiotic is a change in the shape of the relaxation curve as a control for the appearance of cell conglomerates after antibiotic exposure. At concentrations higher than 100 μg/mL, the number of conglomerates decreased but the number of single cells with a small axial ratio increased. As noted earlier, δ*OD* is normalized to the total optical density from single cells and the optical density from conglomerates. As the number of single cells increases and the number of conglomerates decreases, the optical signal magnitude of the mixture increases. The larger is the number of single cells or short chains present in a suspension, the higher is the recorded signal, as compared to the control.

Importantly, when 100–600 μg/mL of ampicillin were used, the character of the recorded signal curve also changed, as compared with the control sample. When the complexes formed from several cells were divided into single cells with a small axial ratio, a bell-shaped component appeared in the graphs. From the obtained data, it can be concluded that the treatment of cells with ampicillin at 100–600 μg/mL caused the conglomerates to break up into single cells or short chains. The weak shift of the peak in the spectrum of the signal indicates a decrease in the electric conductivity of the cytoplasm with an increase in the antibiotic concentration.

A prerequisite for testing any sensor system is to check the results by standard (so-called golden) methods.

### Ampicillin effect on biofilms as observed by phase-contrast microscopy, standard microbiological tests, and confocal laser scanning microscopy

The effect of ampicillin on *P. putida* TSh-18 biofilms was also examined by phase-contrast microscopy by using a Leica LMD 7000 laser microdissection system. This microscopy was chosen because the high numerical aperture of the lenses and the short wavelength of laser radiation make it possible to obtain high-resolution images along the optical and transverse directions. Johnson et al. (2017) showed that it is possible to use a laser microdissection system to control the examination of β*-*lactam effects on bacteria.

**Fig. 5:**
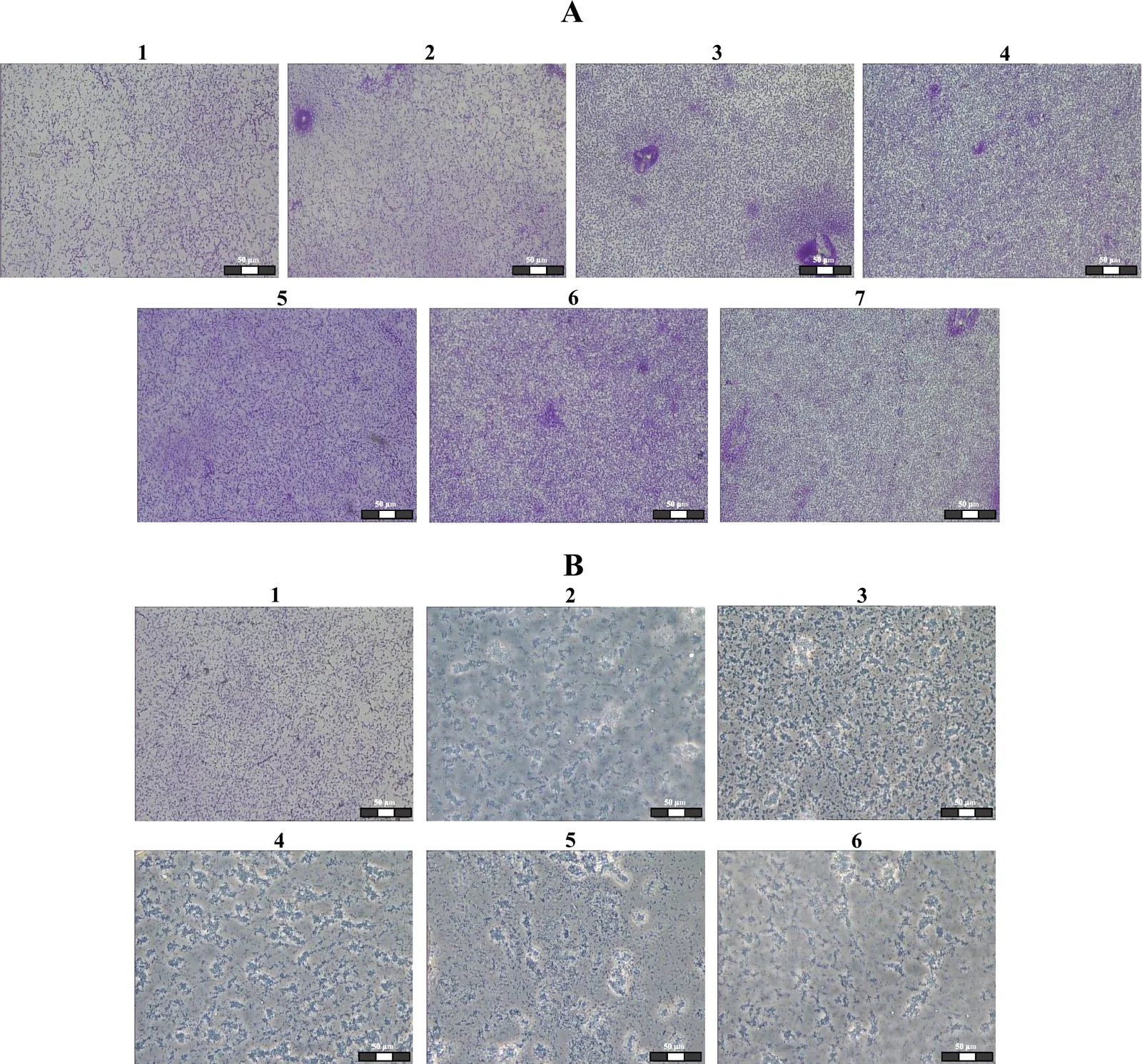
Laser microdissection images of *P. putida* TSh-18 biofilm cells before and after ampicillin treatment. Scale bar: 50 μm. (A) Numbers correspond to the antibiotic concentration: 1 – *control*; 2 – *0.5*; 3 – *1.0*; 4 – *2.5*; 5 – *5.0*; 6 – *10*; 7 – *25* μg/mL. (B) Numbers correspond to the antibiotic concentration: 1 – *control*; 2 – *50*; 3 – *100*; 4 – *250*; 5 – *500*; 6 – *600* μg/mL

Figure 5A shows images of biofilm cells before and after exposure to 0.5–25 μg/mL of ampicillin. Of note, the number of cells treated with 0.5–10 μg/mL of ampicillin did not decrease in the field of view; that is, these concentrations were not lethal to the bacteria. With 25 μg/mL of ampicillin, the number of bacteria in the field of view decreased slightly.

The ampicillin action on sensitive cells causes morphological changes (cell swelling) and damage to the cell wall (Gillespie and McHugh 2010). The change in the magnitude of the signal from *P. putida* TSh-18 biofilms exposed to 25 μg/mL of ampicillin is probably associated with a partial deformation of the cell wall of metabolically active cells, which are located in the outer layers of the biofilm. However, the cells located within the bacterial community remain unaffected, which is consistent with the data of Pamp et al. (2008).

Figure 5B shows the microscopy results for *P. putida* biofilm cells before and after exposure to ampicillin (50–600 μg/mL). With an increase in the ampicillin concentration, the number of cells in the field of view decreased and their shape changed. The bacteria became rounder than those in the control sample. The microscopy data confirmed the optical monitoring results.

The effect of different ampicillin concentrations (0.5–600 μg/mL) on biofilm cell viability was monitored by standard microbiological tests. The results showed that 0.5, 1.0, 2.5, and 10 μg/mL of ampicillin had barely any effect on the viability and biomass of biofilm cells (Figure S4). From 25 μg/mL onward, the number of viable cells decreased significantly, and as a result, total biofilm biomass decreased almost 1.5-fold. Ampicillin was the most inhibitory at 600 μg/mL: the number of CFU/mL decreased almost 10-fold and biofilm biomass decreased 2-fold, which indicates that the formation and functioning of biofilms were disrupted.

At 0.5–10 μg/mL of ampicillin, the bacteria were barely affected. From 25 μg/mL onward, the number of bacteria decreased gradually, in agreement with the optical analysis data (at this concentration, the signal magnitude changed by 17%, as compared with the control). The obtained results clearly show that the magnitude of the signal tends to gradually change with increasing ampicillin concentration.

The strongest effect was obtained with 600 μg/mL of ampicillin. This result may serve as an informational parameter of biofilm sensitivity to this concentration of ampicillin. A similar trend was observed in the comparison of biomass accumulation in biofilms grown under standard conditions and in the presence of 0.5–600 μg/mL of ampicillin.

**Fig. 6:**
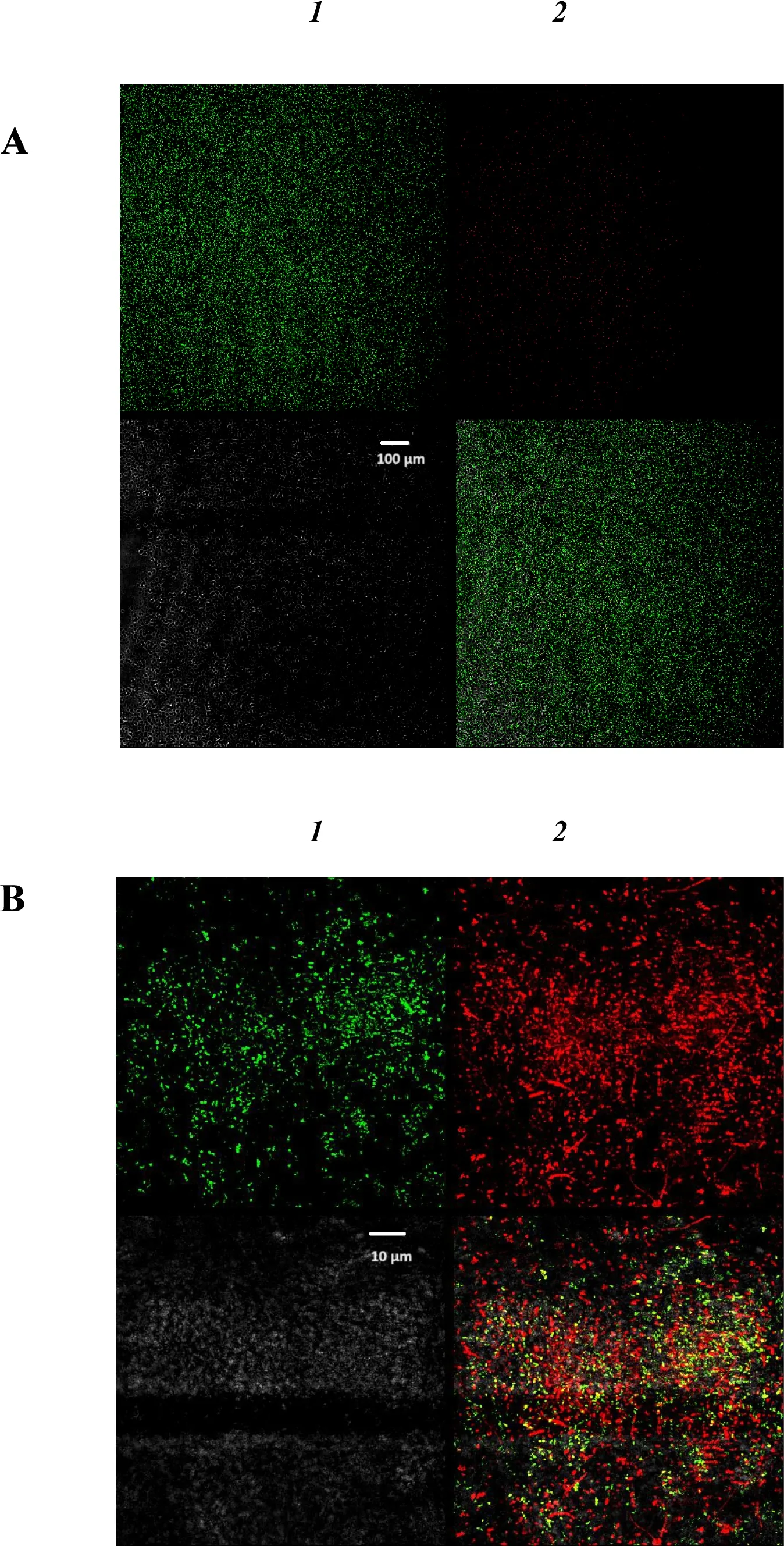
Evaluation of biofilm cell viability by confocal laser scanning microscopy in the absence *(1)* and presence *(2)* of ampicillin (concentrations, 50 and 600 μg/mL), by using LIVE/DEAD BacLight™ kit staining. Representative images of biofilms stained with SYTO 9 dye (green, Ex488/Em 500–550) and propidium iodide (red, Ex561/Em 570–620) (LIVE/DEAD BacLight™ bacterial viability kit; Thermo Fisher Scientific, USA). Biofilm viability at 50 μg/mL of ampicillin (A). Biofilm viability at 600 μg/mL of ampicillin (B). Green stain, live cells; red stain, dead cells

In addition, viability analysis was done by confocal laser scanning microscopy (Fig. 6), by using LIVE/DEAD BacLight™ staining. Comparison of the biofilms grown with and without ampicillin (concentration, 50 and 600 μg/mL) showed that the ratio between living and dead cells changed toward an increase in the number of dead bacteria. Further, in the presence of ampicillin, biofilm structure was disturbed, because the cells were scattered and did not form a biofilm community. This may point to biofilm dispersion in response to the presence of ampicillin in the medium. The obtained data correlate with the results of standard microbiological tests and electro-optical analysis.

## Discussion

Ampicillin targets the bacterial enzymes trans- and carboxypeptidases, collectively called penicillin-binding proteins (PBPs). These enzymes are bound to the cytoplasmic membrane and are involved in the synthesis of peptidoglycan, which ensures the formation of cross-links during the synthesis of the cell wall. The binding of ampicillin to PBPs leads to their inactivation, disruption of cell wall synthesis, cessation of growth, and subsequent cell death. The greater is the affinity of a particular antibiotic for the PBPs of individual microorganisms, the higher is its activity, the stronger is the damaging effect on the membrane, and the more intense is the transport of ions from the cytoplasm. This effect underlies the shift of the peak of the recorded analytic curve to the right (Fig. 4C).

Therefore, the treatment of *P. putida* biofilms with ampicillin leads to changes in the morphology of the bacteria and depends on the antibiotic concentration (dose-dependent effect of the antibiotic on biofilms). These results are in harmony with the data of other research groups for *Pseudomonas* biofilms (Hengzhuang et al. 2011, 2012, 2013, 2014).

Thus, by monitoring changes in the recorded signal of the sensor, it is possible not only to draw preliminary conclusions about the sensitivity of the biofilm to the antibiotic but also to monitor its effect on the biofilm, for example, during antibiotic treatment. This fact permits the sensor response time to be reduced from several days in classical methods to less than 20 min in our approach. In addition, monitoring the effect of an antibiotic on biofilms can provide a conclusion about its applicability to combating the formation and development of biofilms.

Two important points are the monitoring of biofilm growth and the optimization of the analysis procedure when the antibiotic acts on the biofilm. Because the difference between the values of the optical characteristics before and after exposure to the antibiotic serves as an analytic signal, it is possible not to make standard the process of biofilm formation. Nonetheless, in our studies, biofilms were all at the same stage of formation and had similar thicknesses. For optimization of the optical testing of biofilm sensitivity to antibiotics, further experiments are required depending on the time of biofilm growth, the bacterial composition of the biofilm communities, and the antibiotic’s mechanism of action. Changes in the signal enabled us to describe the physical picture of the processes occurring in bacterial biofilms in the presence of ampicillin. The obtained results are in agreement with those of Gimsa (2021), who showed that, according to the Maxwell–Wagner equation, the permittivity of particles actually has a volume character, can be normalized to their number and does not depend on their concentration. The use of an electroanalytic sensor system with a hybrid approach combines the effect of an electric field with an optical result and has several undoubted advantages. The influence of the supporting medium on the accuracy of polarization measurements is insignificant and predictable. The cells are minimally affected by the measuring procedure and retain their vitality.

The main problem in the treatment of biofilm-associated infections is the lack of an adequate standardized method to test biofilm sensitivity to antimicrobial agents (Azeredo et al. 2017; Wiegand et al. 2018).

In view of this, research is underway to develop systems to test the sensitivity of biofilm bacteria. In this work, we have proposed a rapid real-time electroanalytic sensor system with a hybrid approach that permits rapid analysis of the antimicrobial susceptibility of biofilms.

## Supplementary information

ESM 1 (PDF 259 kb)

## Data Availability

All data supporting the findings of this study are available within the paper and its Supplementary Information.
