# Supplementary material for: Sensor system for analysis of biofilm sensitivity to ampicillin
Source: Appl Microbiol Biotechnol. 2024 Jan 24;108:172. doi: 10.1007/s00253-023-12831-7 (PMC10808281; doi:10.1007/s00253-023-12831-7)
Supplement: Supplementary file 1 [file 253_2023_12831_MOESM1_ESM.pdf]

## Supporting Information

# Sensor system for analysis of biofilm sensitivity to ampicillin

Olga I. Guliy [a, *], Stella S. Evstigneeva [a], Alexander A. Shirokov [a], Victor D. Bunin [b]

[a] *Institute of Biochemistry and Physiology of Plants and Microorganisms – Subdivision of the Federal State Budgetary Research Institution Saratov Federal Scientific Centre of the Russian Academy of Sciences (IBPPM RAS), Saratov 410049, Russia*

[b] *EloSystems GbR, Berlin, 13407, Germany*

## Table of contents

**Figure S1** The main scheme of the sensor measurement before and after antibiotic treatment

**Figure S2** Changes in the electro-optical signal in cell orientation ($S_1$) and disorientation ($S_2$)

**Figure S3** Polarization of boundaries between cell structures under the effect of an electric field

**Figure S4** Changes in the viability of *P. putida* TSh-18 biofilms after treatment with various ampicillin concentrations

**Table S1** Coefficient values for the polynomial function for calculating the average cell size on the basis of $S_{relax}$ on the selected time scale **T**

[*] Corresponding author. Institute of Biochemistry and Physiology of Plants and Microorganisms – Subdivision of the Federal State Budgetary Research Institution Saratov Federal Scientific Centre of the Russian Academy of Sciences (IBPPM RAS), Saratov 410049, Russia. *E-mail address:* guliy_olga@mail.ru (O.I. Guliy).

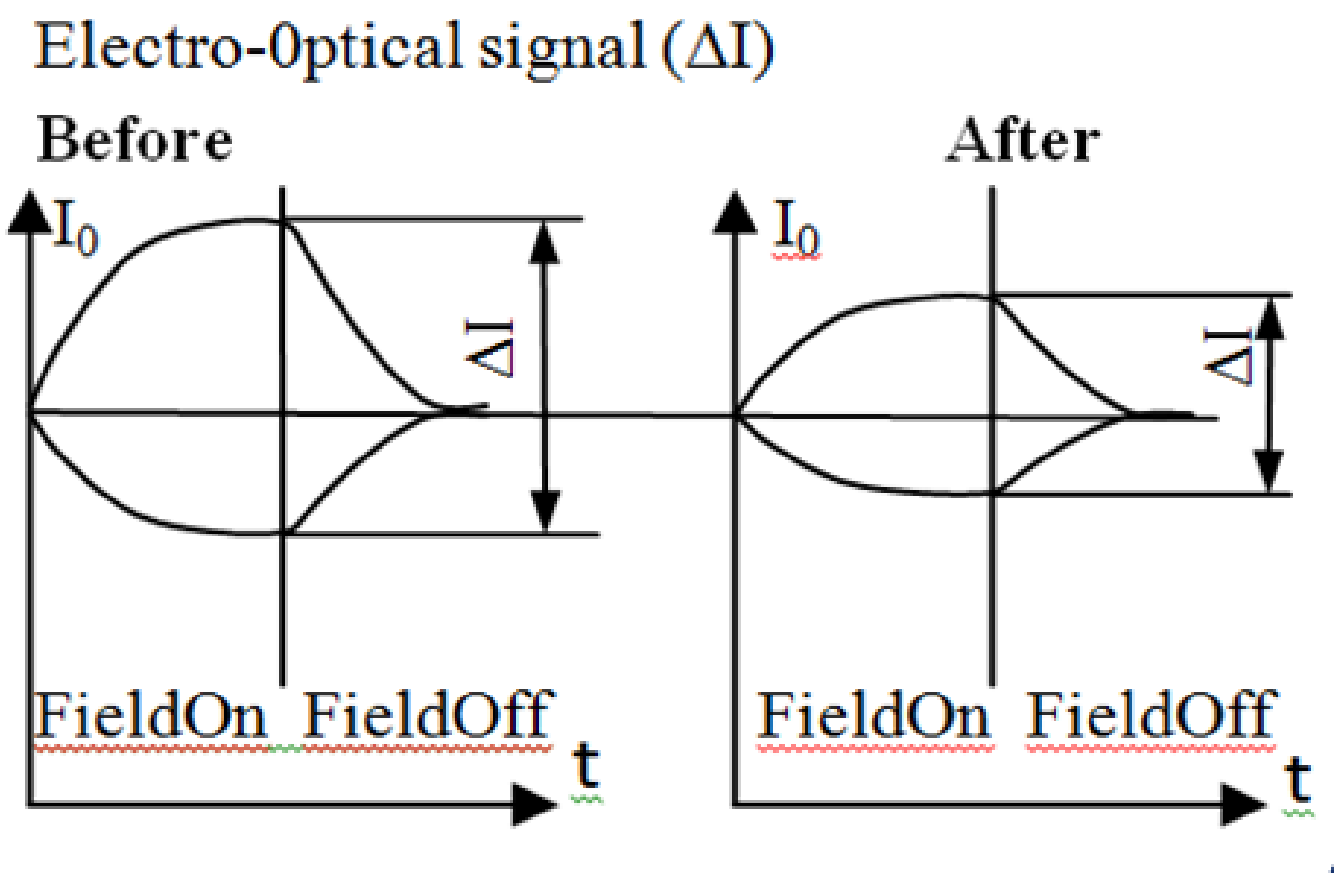


**Figure S1** The main scheme of the sensor measurement before and after antibiotic treatment

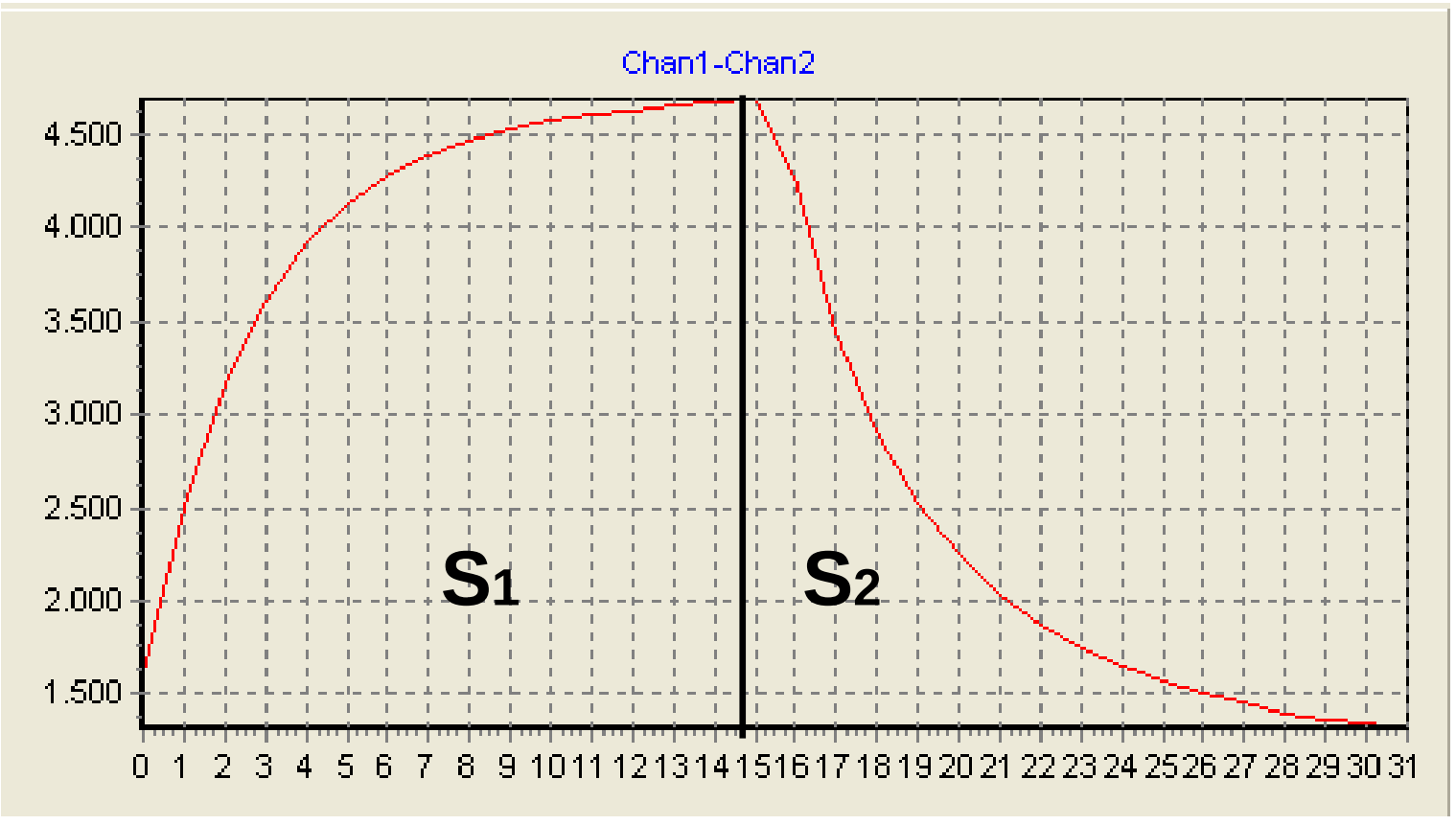


**Figure S2** Changes in the electro-optical signal in cell orientation ($S_1$) and disorientation ($S_2$)

**Table S1** Coefficient values for the polynomial function for calculating the average cell size on the basis of $S_{relax}$ on the selected time scale ***T***

| Y = A+B*X^C (KWD = $S_{relax}$)<br>Cell size, µm = A+B*($S_{relax}$)^C | | | | |
|---|---|---|---|---|
| Time | | A | B | C |
| T4 | 2.0s | 0.8 | 103 | - 3.2 |
| T5 | 3,0 s | 0.8 | 105 | -2.8 |
| T6 | 4.0 s | 0.8 | 106 | -2.52 |
| T7 | 6.0 s | 0.8 | 112 | -2.26 |
| T8 | 9.0 s | 0.8 | 124 | -2.05 |
| T9 | 12.0 s | 0.8 | 148 | -1.91 |
| T10 | 18.0 s | 0.8 | 166 | -1.81 |

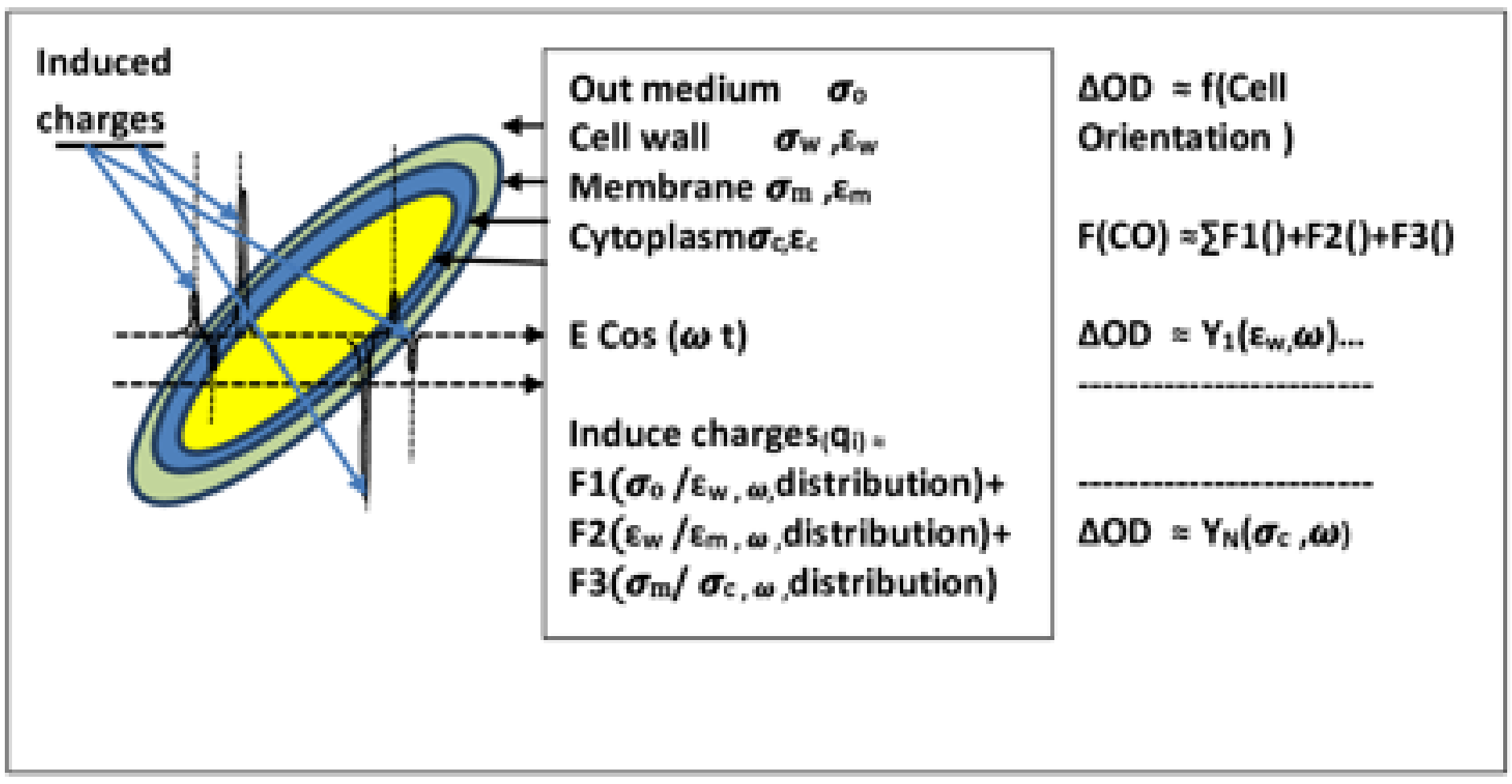


**Figure S3** Polarization of boundaries between cell structures under the effect of an electric field

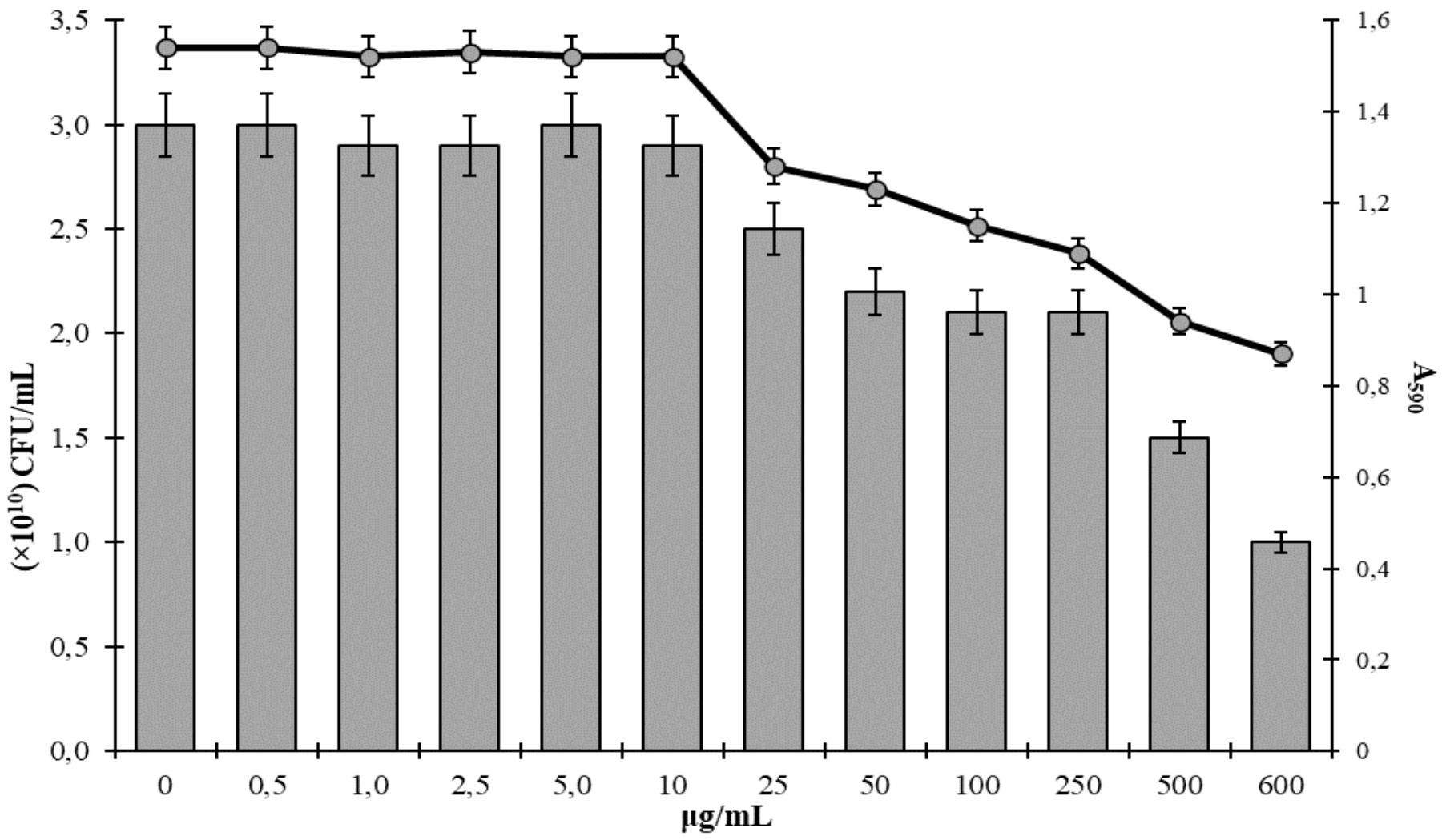


**Figure S4** Changes in the viability of *P. putida* TSh-18 biofilms after treatment with various ampicillin concentrations
